# Synthesis and characterization of aluminium substituted calcium hexaferrite

**DOI:** 10.1016/j.heliyon.2020.e03186

**Published:** 2020-01-17

**Authors:** V.S. Shinde, S.G. Dahotre, L.N. Singh

**Affiliations:** a Department of Physics, K.E.S. Anandibai Pradhan Science College, Nagothane M.S., India; b Department of Physics, Dr. Babasaheb Ambedkar Technological University, Lonere M.S., India

**Keywords:** Materials science, Nanomaterials, Materials application, Materials characterization, Materials processing, Materials structure, Materials synthesis, Calcium hexaferrite, Sol gel auto combustion method, Lattice volume, Saturation magnetisation, Coercivity

## Abstract

Al substituted M type Ca hexaferrite with composition CaAl_x_Fe_12-x_O_19_ (x = 0, 0.2, 0.4, 0.6, 0.8) were synthesized by sol gel auto combustion method. The prepared samples were characterized by XRD, SEM, FTIR and VSM. X ray diffraction study shows that the with increasing aluminum ion concentration lattice parameter a decreases from 5.87 Å to 5.83 Å while the lattice parameter c decreases from 22.15 Å to 22.00 Å are well within the range of M type of hexaferrite. The crystallite size of the particles decreases from 74.36nm to 62.12nm are suitable for magnetic recording. Morphology of the particles from SEM images was hexagonal platelet. The absorption band between 580 and 440cm^−1^ in FTIR confirm the formation of hexaferrite. The magnetic properties of the samples changes with Al ion substitution make the material suitable for low density longitudinal and perpendicular magnetic recording.

## 1 Introduction

The consumer demand for hard disk drive is rapidly increasing because of strong growth of information. There are two magnetic recording techniques in hard disk drive, the ancient longitudinal magnetic recording and recent perpendicular recording. In perpendicular recording the size of the disk is reduced which increases the storage capacity. If we get the small particle size and high value of coercivity the material is suitable for perpendicular recording. Calcium ferrite particles are considered as a promising material for high density magnetic recording, Single domain Ca ferrite particles without substitution have large coercivity. The coercivity can be changed by substitution with other diamagnetic or paramagnetic cations [1].

Hexagonal ferrites are extensively used in permanent magnets, magnetic recording media, data storage devices, microwave components, high frequency circuits and operating devices due to large uniaxial magneto crystalline anisotropy, high saturation magnetisation, excellent chemical stability, corrosion resistivity and low price [2].

Hexagonal ferrite possesses the hexagonal structure and is of the type of hard ferrite. Depending on chemical formulae and crystal structure hexagonal ferrites are divided into five types as M, W, X, Y and Z [3]. The general formula of M type hexaferrite is MFe_12_O_19_ where M is usually divalent metal ion such as barium, strontium, calcium or Lead [4]. The electronic configuration of Sr, Ba and Ca are similar and lies in the same group of periodic table [5]. Abundances of Calcium is more than strontium and barium on the earth, and its price is relatively cheaper. Besides this calcium hexaferrites have been less studied and has magnetic properties comparable to barium and strontium hexaferrite [6]. The unit cell of M type of hexaferrite is composed of the stacking sequence SRS*R*, where S* and R* blocks are rotation of S and R blocks at 180° about the hexagonal *c*-axis. Within S block, there are three interstitial sites, one octahedral 2a site occupied by spin-up Fe^3+^ ion and two tetrahedral 4f_1_ sites occupied by spin-down Fe^3+^ ions. Within R block there are three interstitial sites, two octahedral 4f_2_ sites occupied by spin-up Fe^3+^ ions and one trigonal bi-pyramidal 2b site occupied by spin-up Fe^3+^ ion. There are three interstitial octahedral (12k) sites within each R-S interface layer, which are occupied by spin-up Fe^3+^ ions [7].

The performance of M-type hexaferrites must be improved by substitution with trivalent ions such as La^3+^, Al^3+^, Sm^3+^, Bi^3+^, Nd^3+^, Cr^3+^ etc., [8].

Hexagonal ferrites were prepared by different methods such as chemical co-precipitation, low-temperature combustion, sol–gel, solid-state reaction, micro emulsion and reverse micro emulsion [9].

Ch.Mamatha et al., were synthesized Al substituted nano calcium hexaferrite CaAl_x_Fe_12-x_O_19_ (x = 3, 4) by Solution combustion technique using metal nitrates as oxidants and ODH as reducing agents. It has been observed that with increasing Al ion concentration the crystallite size and lattice volume decreases. Coercivity and remanent magnetisation increases while saturation magnetisation decreases with increasing Al ion concentration Increase in coercivity and retentivity make these materials as hard ferrite materials [4].

S.R. Gawali and et al., were synthesized the Al substituted nano calcium hexaferrite CaAl_x_Fe_12-x_O_19_ (x = 0, 2, 4) by sol-gel auto-combustion technique using urea as a fuel. It has been observed that the lattices parameters and lattice volume decreases with increasing Al ion concentration. The calculated values of a and c is found to be 5.8 Å and 22.1 Å respectively [10].

In current research, M-type calcium hexaferrite with formula CaAl_x_Fe_12-x_O_19_ were synthesized by sol-gel auto-combustion method. It is seen that among all methods sol-gel auto combustion method is superior method. This method is simple, safe and rapid process having advantages of high homogeneity, high purity, time saving and ultra fine powers [11, 12, 13].

Al ion substitution in calcium hexaferrite above concentration x = 1 is more studied while Al ion concentration below concentration 1 it is not studied. Hence an attempt has been made to study the effect of Al substitution below the concentration 1 and to study its structural and magnetic properties.

## 2 Materials and methods

The samples of M-type Al substituted Ca hexaferrites with formula CaAl_x_Fe_12-x_O_19_ (x = 0, 0.2, 0.4, 0.6, 0.8) were synthesized by sol gel auto-combustion method.

Stoichiometric amount of A.R. grade calcium nitrate Ca(NO_3_)_2_ .4H_2_O, iron nitrate Fe (NO_3_)_3_ .9H_2_O, aluminium nitrate Al(NO_3_)_3_ .9H_2_O and citric acid were dissolved in double filtered distilled water. The ratio of citric acid to metal nitrate ions was 1:1. The solution was neutralized to pH 7 by adding liquor ammonia. Then the solution was heated at 80 °C on a hot plate with continuous stirring. After evaporation of water the solution became a viscous brown gel. As temperature increases dried gel burnt in a self propagation combustion manner to form a loose powder. The powder was grinded in a pestle and mortar to form fine particles. The fine particles thus obtained were calcinated at 950 °C in electric furnace for 4 h to obtain ferrite nano particles.

The structural characterizations of the samples were performed by Panalytical X’ Pert Pro diffractometer with Cu-K_α_ radiation of wavelength 1.54184 Å. The Quanta 200 FEG scanning electron microscope was used to observe the morphology of the particles. Fourier Transform Infra-Red (FT-IR) spectra were recorded using Schimadzu Perkin-Elmer Spectrum FT-IR instrument with KBr pellets in the range 4000–450 cm^−1^. Magnetic Properties of the samples were measured using Lakeshore VSM 7410 model.

## 3 Results and discussion

**Figure 1:**
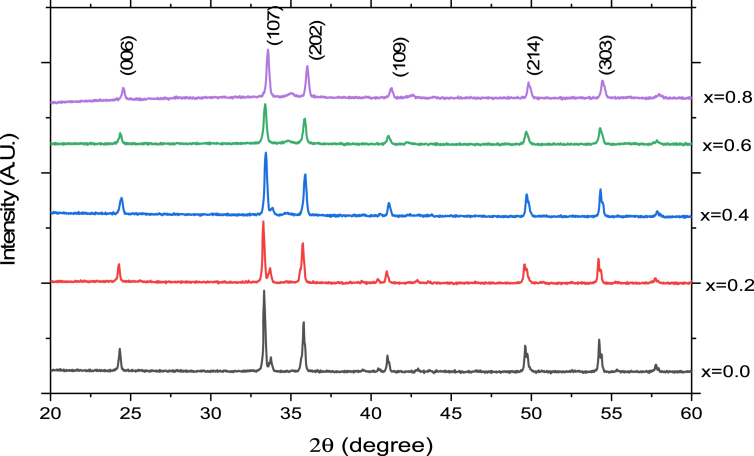
XRD pattern of CaAl_x_Fe_12-x_O_19_.

Figure 1 shows XRD patterns of M type hexaferrite with composition CaAl_x_Fe_12-x_O_19_ (x = 0, 0.2, 0.4, 0.6, 0.8). The X-ray diffraction patterns shown in Figure 1 are used to determine the structural information of the processed materials.

The observed XRD patterns are indexed with the standard JCPDS pattern (file no. 49-1586) of the M-type hexaferrite. The highest intensity diffraction peak of all the samples were found at (107) orientation. There was minor shift in peak position with increase in Al^3+^ ion concentration. The XRD pattern consists of standard reflecting planes (006), (107), (202), (109), (214) and (3.0,3) confirm that the prepared samples belongs to M type hexaferrite.

The crystallite size (D) was determined from the position of the highest diffraction peak using the well known Scherrer equation [14].

(1) $$\text{D}=\frac{K\lambda }{\beta cos\theta }$$

where β is angular line width at high maximum intensity and θ is the Bragg's angle of maximum intensity peak.

The lattice constants (a and c) and lattice Volume of unit cell (V_cell_) were calculated by using following equations

(2) $$\frac{1}{d^{2}}=\frac{4}{3}\left(\frac{h^{2}+hk+k^{2}}{a^{2}}\right)+\frac{l^{2}}{c^{2}}$$

(3) $$V_{cell}=\frac{\sqrt{3}}{2}a^{2}\text{c}$$

The structural properties of the samples are given in Table 1.

**Table 1:** Structural parameters of Al substituted Ca Hexaferrite.

Sample	D (nm)	Lattice Parameters		c/a	V (${Å}^{3}$)
		a (Å)	c (Å)		
CaFe_12_O_19_	74.36	5.87	22.15	3.77	660.9011
CaAl_0.2_Fe_11.8_O_19_	74.31	5.87	22.19	3.77	663.9358
CaAl_0.4_Fe_11.6_O_19_	70.03	5.85	22.09	3.77	655.5814
CaAl_0.6_Fe_11.4_O_19_	68.41	5.85	22.11	3.77	657.3745
CaAl_0.8_Fe_11.2_O_19_	62.12	5.83	22.00	3.77	648.0615

**Figure 2:**
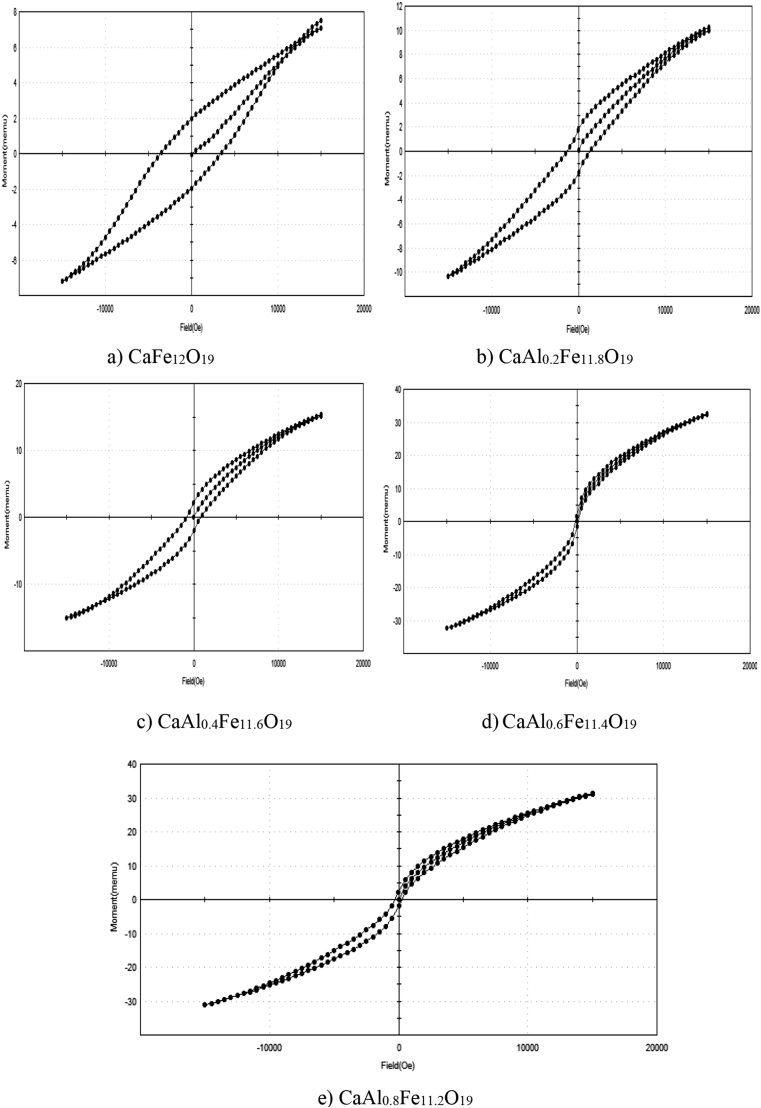
Hysteresis loop of CaAl_x_Fe_12-x_O_19_ magnetic powder a) CaFe_12_O_19_ b)CaAl_0.2_Fe_11.8_O_19_ c) CaAl_0.4_Fe_11.6_O_19_ d) CaAl_0.6_Fe_11.4_O_19_ e) CaAl_0.8_Fe_11.2_O_19_.

It was observed that the lattice parameters (a and c), lattice volume and crystallite size decreases with increase in Al ion concentration. The crystallite sizes for all the samples are in nano range, Typical room-temperature hysteresis loops for samples Ca Al_x_Fe_12-x_O_19_ (x = 0, 0.2, 0.4, 0.6, 0.8) prepared using sol gel auto combustion method are shown in Figure 2 and relevant data are presented in Table 2.

**Table 2:** Magnetic parameters of Al substituted Calcium Hexaferrite.

Sample	M_s_ (emu/gm)	H_c_ (Oe)	M_r_ (emu/gm)	M_r_/M_s_
CaFe_12_O_19_	0.49	3493.10	0.13	0.26
CaAl_0.2_Fe_11.8_O_19_	0.69	1343.40	0.11	0.16
CaAl_0.4_Fe_11.6_O_19_	1.02	843.50	0.14	0.13
CaAl_0.6_Fe_11.4_O_19_	2.16	138.28	0.10	0.05
CaAl_0.8_Fe_11.2_O_19_	2.08	235.10	0.12	0.06

**Figure 3:**
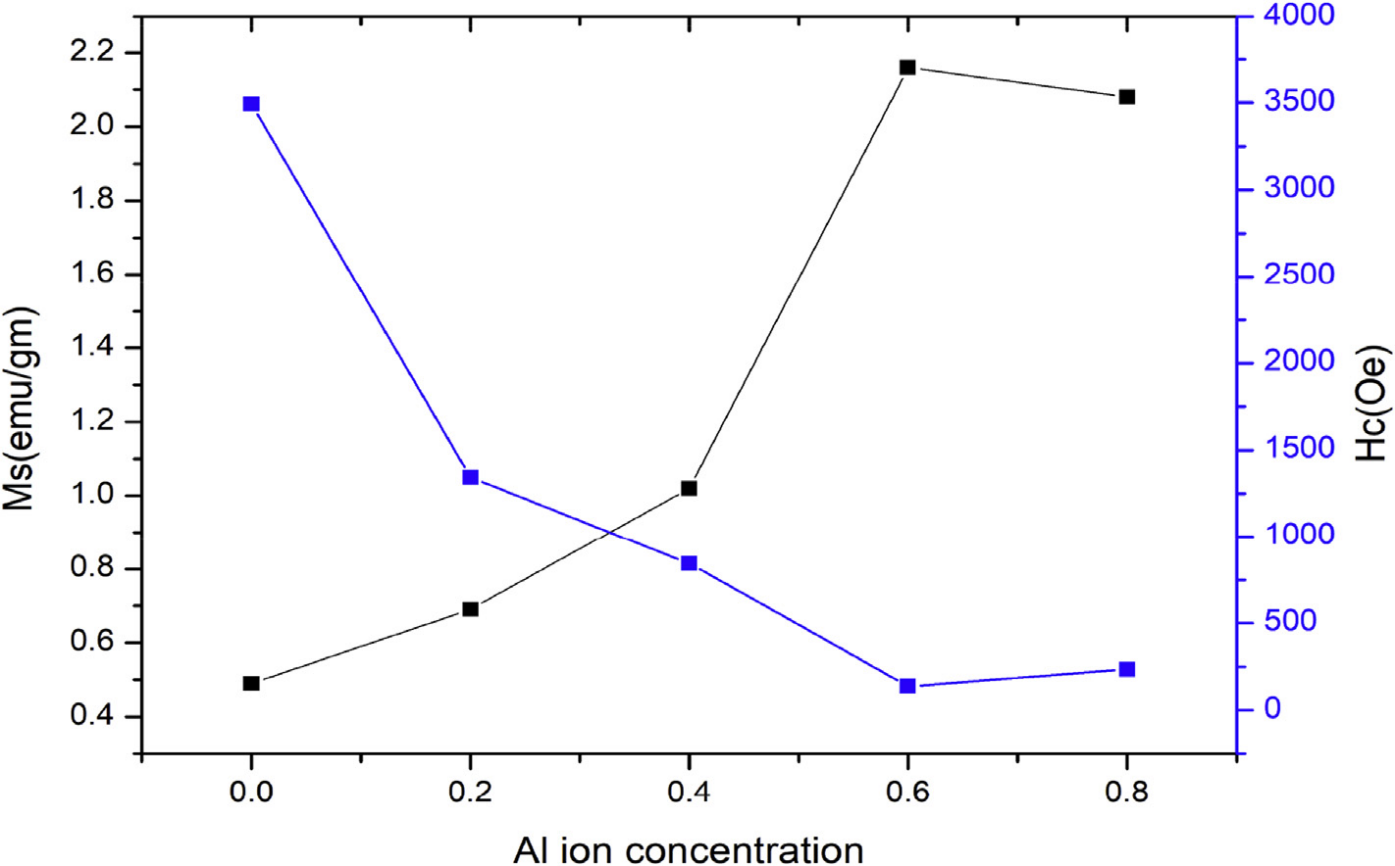
Plot of M_s_ and H_c_ against various Ni ion concentrations.

The effect of Al^3+^ ion substitution on saturation magnetisation and coercivity are graphically represented in Figure 3.

Magnetic parameters obtained from hysteresis loop shown in Table 2 and Figure 3 shows that, as Al^3+^ ion concentration increases the saturation magnetisation increases from x = 0 to x = 0.6, while the coercivity decreases in this range. Above the concentration x = 0.6, the saturation magnetisation decreases while the coercivity increases.

**Figure 4:**
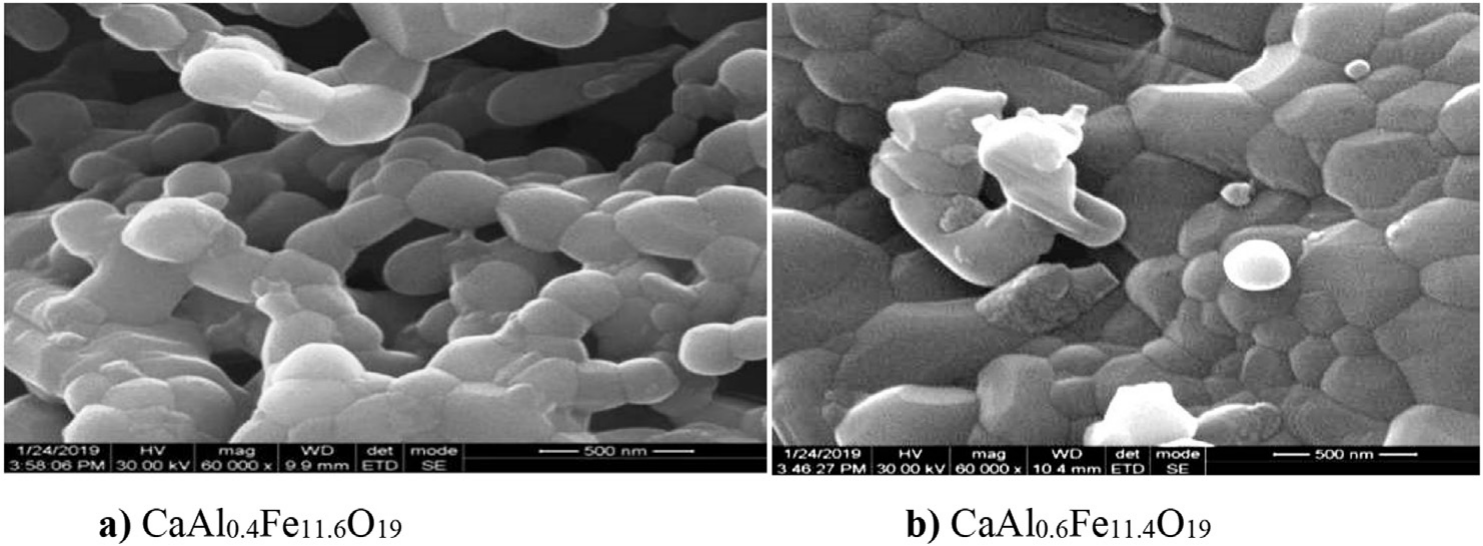
FE-SEM micrographs of calcium hexaferrite a**)** Ca Al_0.4_Fe_11.6_O_19_ b) CaAl_0.6_Fe_11.4_O_19_.

The micrographs of the Ca-hexaferrite nanoparticales with chemical composition CaAl_0.2_Fe_11.8_O_19_ and CaAl_0.4_Fe_11.6_O_19_ calcinated at 950 °C is displayed in Figure 4.

SEM micrographs shows that, the sample have fine grains having hexagonal platelet structure and grain size decreases with increasing Al ion substitution. The observed grain size is in nanometer range.

**Figure 5:**
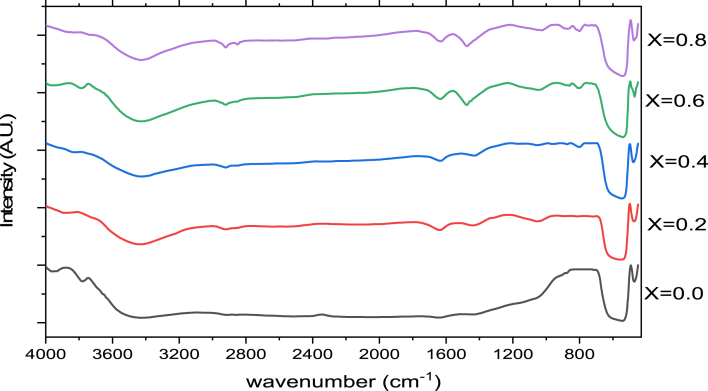
FT-IR spectra of Al substituted calcium hexaferrite.

FT-IR spectra of Al substituted calcium hexaferrite for various Al concentrations are shown in Figure 5.

The X-ray crystallographic study of all five samples shows that, the lattice volume decreases with increase in aluminum ion concentration. The substituted Al^3+^ (0.54 Å) ion replaces Fe^3+^ (0.65 Å) ion in CaAl_x_Fe_12-x_O_19_. Due to the difference in the ionic radii of Al^3+^ and Fe^3+^ ion the lattice volume decreases with increasing Al^3+^ ion concentration [14]. The lattice constants a and c decreases with increasing Al^3+^ ion concentration. For the concentration x = 0.2 and x = 0.6 the lattice parameter c increases which is likely associated with defects and lattice distortion. The crystal axis contraction (c/a) is nearly constant. The reduction in cell dimensions is due to the solubility of Al^3+^ ions in the M type hexaferrite [15].

According to Verstegen and Stevels, the M-type structure can be considered, if c/a ratio is lower than 3.98. The c/a ratios were calculated for the entire prepared sample ranges from 3.60 to 3.64 are well within the ratio range of M-type structures [8].

In the crystal structure of M type hexaferrite Fe^3+^ ions are distributed in five different sites, three octahedral sites (12k, 4f_2_ and 2a), one tetrahedral site (4f_1_) and the last one is trigonal bipyramidal site (2b) [16]. Fe^3+^ ions distributed on 12k, 2a and 2b sites have spin up electronic configuration, while those located on 4f_1_ and 4f_2_ sites have spin down electronic configuration [17]. Fe^3+^ ions having magnetic moment of 5μB are substituted by Al^3+^ ions having magnetic moment of 0 μB. The improvement in saturation magnetisation is due to the replacement of Fe^3+^ ions in the spin down state [18].

The literature review result shows that, the substituted Al^3+^ ions have the preference to occupy 12k, 2a and 4f_1_ sites [19]. For lower Al^3+^ ion concentration (x = 0.0 to x = 0.6), Al^3+^ ions enter 4f_1_ site. 4f_1_ site has spin down electronic configuration. This reduces the electrons with spin down electronic configuration and increases the electrons in upward spin direction. The result is that resultant magnetic moment and saturation magnetisation increases. For higher Al^3+^ ion concentration (above x = 0.6), Al^3+^ ions enter 12K site [20]. 12K site has spin up electronic configuration. This increases the electrons with spin down electronic configuration and decreases the electrons in upward spin direction. With increasing the substitution of the Al^3+^ ions (x > 0.6) instead of Fe^3+^ ions, the the super exchange interaction Fe^3+^-O-Fe^3+^ is also reduced. The combined result is that resultant magnetic moment and saturation magnetisation decreases.

The coercivity of particles is determined by using magneto crystalline anisotropy constant K and saturation magnetization [3].

(4) $$H_{c}=\frac{2K}{\mu_{0}M_{s}}$$

where K is Magneto crystalline anisotropy constant.

The coercivity (H_c_) of samples decreased with an increase in Al^3+^ ion content is due to the decrease of magneto crystalline anisotropy [3].

From the magnetization curve the squareness ratio (M_r_/M_s_) is less than 0.5. Such a material is good for recording medium [20]. For longitudinal magnetic recording medium high coercivity up to 600 Oe is required. For the coercivity value above 1200 Oe, the material can be used for the perpendicular recording media [21]. The obtained values of coercivity are such that the synthesized material will be suitable for longitudinal and perpendicular recording media.

FTIR spectra indicate the characteristics bands in the range 3200–3600cm^−1^ is the O–H stretching vibration of water molecule. The absorption band due to the bending mode of H–O–H molecule around 1630cm^−1^ is diagnostic of the presence of water of hydration in all the samples. Stretching peak at 1027 cm^−1^ indicates C–O while the bending peak at 1474cm^−1^ indicates C–H vibration. Stretching peak at 541 cm^−1^ and 474 cm^−1^ indicates existence of metal-oxygen vibration mode of hexaferrite structure including octahedral and tetrahedral sites respectively. The absorption band between 580 and 440cm^−1^ confirm the formation of hexaferrite [22].

## 4 Conclusion

In the present research Al substituted Ca hexaferrite were synthesized by auto combustion method. The XRD data confirmed the formation of single phase magnetoplumbite M type hexaferrite. The lattice parameters a and c support the confirmation. The FTIR data also confirmed the formation of hexaferrite. SEM micrograph gives the grain size in nano range. With the substitution of Al ion in Ca hexaferrite the magnetic properties such as saturation magnetisation, coercivity, remanent magnetisation changes. The observed results suggest that these synthesized hexaferrites have potential applications for longitudinal and perpendicular recording media.

## Declarations

### Author contribution statement

V. S.Shinde, S.G. Dahotre & L.N.Singh: Conceived and designed the experiments; Performed the experiments; Analyzed and interpreted the data; Contributed reagents, materials, analysis tools or data; Wrote the paper.

### Funding statement

This research did not receive any specific grant from funding agencies in the public, commercial, or not-for-profit sectors.

### Competing interest statement

The authors declare no conflict of interest.

### Additional information

No additional information is available for this paper.
